# Vascular endothelial growth factor-A is an Immunohistochemical biomarker for the efficacy of bevacizumab-containing chemotherapy for duodenal and jejunal adenocarcinoma

**DOI:** 10.1186/s12885-021-08724-5

**Published:** 2021-08-31

**Authors:** Takahiro Amano, Hideki Iijima, Shinichiro Shinzaki, Taku Tashiro, Shuko Iwatani, Mizuki Tani, Yuriko Otake, Takeo Yoshihara, Aya Sugimoto, Satoshi Egawa, Shinjiro Yamaguchi, Kazuo Kinoshita, Manabu Araki, Motohiro Hirao, Yuko Sakakibara, Satoshi Hiyama, Hiroyuki Ogawa, Koji Nagaike, Jun Murata, Masato Komori, Yorihide Okuda, Takashi Kizu, Yoshiki Tsujii, Yoshito Hayashi, Takahiro Inoue, Hidekazu Takahashi, Tsunekazu Mizushima, Eiichi Morii, Tetsuo Takehara

**Affiliations:** 1 Department of Gastroenterology and Hepatology, Osaka University Graduate School of Medicine, 2-2 Yamadaoka, Suita, Osaka, 565-0871 Japan; 2 Department of Internal Medicine, Osaka Police Hospital, Osaka, Japan; 3 Department of Gastroenterology, Toyonaka Municipal Hospital, Toyonaka, Osaka, Japan; 4 Department of Gastroenterology, Kansai Rosai Hospital, Amagasaki, Hyogo Japan; 5 Department of Gastroenterology, Otemae Hospital, Osaka, Japan; 6 Department of Gastroenterology, National Hospital Organization Osaka Minami Medical Center, Kawachinagano, Osaka, Japan; 7 Department of Gastroenterology and Hepatology, Osaka Rosai Hospital, Sakai, Osaka, Japan; 8 Department of Gastroenterology, National Hospital Organization Osaka National Hospital, Osaka, Japan; 9 Department of Gastroenterology, Japan Community Healthcare Organization Osaka Hospital, Osaka, Japan; 10 Department of Gastroenterology, Nishinomiya Municipal Central Hospital, Nishinomiya, Hyogo Japan; 11 Department of Gastroenterology and Hepatology, Suita Municipal Hospital, Suita, Osaka, Japan; 12 Department of Gastroenterology, Higashiosaka City Medical Center, Higashiosaka, Osaka, Japan; 13 Department of Gastroenterology, Hyogo Prefectural Nishinomiya Hospital, Nishinomiya, Hyogo Japan; 14 Department of Gastroenterology, Saiseikai Senri Hospital, Suita, Osaka, Japan; 15 Department of Gastroenterology, Yao Municipal Hospital, Yao, Osaka, Japan; 16 Department of Gastroenterological Surgery, Osaka University Graduate School of Medicine, Suita, Osaka, Japan; 17 Department of Pathology, Osaka University Graduate School of Medicine, Suita, Osaka, Japan

**Keywords:** Small bowel adenocarcinoma, Duodenal and jejunal adenocarcinoma, VEGF-A, Immunohistochemical expressions, Bevacizumab

## Abstract

**Background:**

The efficacy and safety of bevacizumab-containing chemotherapy for patients with metastatic duodenal and jejunal adenocarcinoma (mDJA) are unclear. The present study aimed to evaluate the efficacy of bevacizumab and to explore immunohistochemical markers that can predict the efficacy of bevacizumab for patients with mDJA.

**Methods:**

This multicentre study included patients with histologically confirmed small bowel adenocarcinoma who received palliative chemotherapy from 2008 to 2017 at 15 hospitals. Immunostaining was performed for vascular endothelial growth factor-A (VEGF-A), TP53, Ki67, β-catenin, CD10, MUC2, MUC5AC, MUC6, and mismatch repair proteins.

**Results:**

A total of 74 patients were enrolled, including 65 patients with mDJA and 9 with metastatic ileal adenocarcinoma. Patients with mDJA who received platinum-based chemotherapy with bevacizumab as first-line treatment tended to have a longer progression-free survival and overall survival than those treated without bevacizumab (*P* = 0.075 and 0.077, respectively). Multivariate analysis extracted high VEGF-A expression as a factor prolonging progression-free survival (hazard ratio: 0.52, 95% confidence interval: 0.30–0.91). In mDJA patients with high VEGF-A expression, those who received platinum-based chemotherapy with bevacizumab as a first-line treatment had significantly longer progression-free survival and tended to have longer overall survival than those treated without bevacizumab (*P* = 0.025 and *P* = 0.056, respectively), whereas no differences were observed in mDJA patients with low VEGF-A expression.

**Conclusion:**

Immunohistochemical expression of VEGF-A is a potentially useful biomarker for predicting the efficacy of bevacizumab-containing chemotherapy for patients with mDJA.

**Supplementary Information:**

The online version contains supplementary material available at 10.1186/s12885-021-08724-5.

## Background

Although the small bowel comprises 75% of the total length and more than 90% of the mucosal surface area of the gastrointestinal tract [1, 2], small bowel adenocarcinoma (SBA) as a primary tumour location is very rare, comprising only 1 to 3% of all gastrointestinal cancers. SBA accounts for 30 to 40% of all small bowel cancers and the annual incidence of SBA is approximately 3.9 per million persons in the United States [1] and 5.0 per million persons in Europe [3]. Because of the delayed manifestation of symptoms and the difficulty screening the entire small bowel by conventional esophagogastroduodenoscopy and colonoscopy [1, 4–7], 30 to 35% of SBA is diagnosed with distant metastases [8–10].

We recently demonstrated that bevacizumab in combination with platinum-based chemotherapy is effective and well-tolerated for metastatic SBA (mSBA) [11], consistent with other reports [12–15]. Furthermore, Legue et al. [15] reported that bevacizumab is effective for metastatic ileal adenocarcinoma (mIA), but it has remained unclear whether bevacizumab is also effective for metastatic duodenal and jejunal adenocarcinoma (mDJA). Bevacizumab is an anti-vascular endothelial growth factor (VEGF) monoclonal antibody that binds to VEGF-A and prevents its binding to VEGF receptors on endothelial and cancer cells [13]. Overman et al. [16] reported that characterisation of VEGF-A expression has potential benefit for a VEGF-targeted therapeutic strategy in SBA. Rohrberg et al. [17] reported that immunohistochemical expression of VEGF-A could be a biomarker for the efficacy of bevacizumab in upper gastrointestinal cancers, including metastatic gastric cancer (GC). The potential use of VEGF-A expression as a biomarker for the efficacy of bevacizumab for mDJA, however, has not yet been evaluated.

Mucinous immunophenotypic classification (i.e., intestinal [I]-type, gastrointestinal [GI]-type, gastric [G]-type, or null [N]-type) using CD10, MUC2, MUC5AC, and MUC6 have been investigated in GC and colorectal cancer (CRC). The mucinous immunophenotype is reported to be a prognostic factor in GC [18] and useful for evaluating the biological behaviour and tumorigenesis of CRC [19, 20]. The usefulness of this classification for evaluating the prognosis or tumorigenesis in mSBA, however, has not been investigated. Furthermore, immunohistochemical investigation, including VEGF-A expression and mucinous immunophenotypic classification, regarding the use of bevacizumab in patients with mDJA has not been performed. The aim of the present study was to comprehensively analyse immunohistochemical expression, including VEGF-A expression, and to explore the usefulness of immunohistochemical expression for determining the first-line chemotherapy, especially in combination with bevacizumab, for patients with mDJA.

## Methods

### Patients

This was a retrospective multicentre study. From January 2008 to December 2017, we enrolled patients over 16 years of age who were histologically diagnosed with adenocarcinoma of the duodenum (excluding the ampulla of Vater), jejunum, or ileum, and had received palliative chemotherapy for unresectable disease or disease recurrence with residual specimens sufficient for immunohistochemical staining at 15 hospitals participating in the Osaka Gut Forum. This study was performed in accordance with the Declaration of Helsinki, and the ethics committees of each individual institution approved the study. Written informed consent was waived by the ethics committees by providing participants the opportunity to opt out of the study.

### Data collection

The following data were obtained from the medical records at each institution: patient characteristics (age, sex, Eastern Cooperative Oncology Group performance status [PS]) [21], primary tumour locations (duodenum excluding the ampulla of Vater, jejunum, or ileum), histological type (differentiated/undifferentiated) [22], tumour biomarker level (serum carcinoembryonic antigen [CEA] and carbohydrate antigen 19–9 [CA19–9]), the number of metastatic organs, and metastatic site (liver, lung, lymph node or peritoneal dissemination). Best response to chemotherapy was evaluated according to the Response Evaluation Criteria in Solid Tumours (version 1.1) [23]. The National Cancer Institute Common Terminology Criteria (version 4.0) [24] was used to evaluate the toxicity of therapeutics. Progression-free survival (PFS) was defined as the duration from the initiation of chemotherapy until the date of disease progression. Overall survival (OS) was defined as the duration from the initiation of chemotherapy until death, loss of follow-up, or current date. Surviving patients were censored on their last follow-up date.

### Treatment

The patients were divided into 3 groups according to the first-line chemotherapy regimen based on the use of bevacizumab and chemotherapy with fluoropyrimidine and platinum: Bevacizumab+ Platinum Group, patients who received bevacizumab in combination with CAPOX or modified FOLFOX6 (mFOLFOX6); Platinum Group, patients who received fluoropyrimidine and platinum without bevacizumab; Monotherapy Group, patients who received monotherapy with fluoropyrimidine or other because they were considered not able to tolerate combination therapy due to advanced age, low PS, etc. These treatments were generally repeated until disease progression, unacceptable toxicity, or a patient’s request to terminate treatment. The chemotherapy regimens for each group were as follows:

- Bevacizumab+ Platinum Group.
  1. Combined use of bevacizumab with CAPOX: 7.5 mg/kg bevacizumab and oxaliplatin (130 mg/m^2^) intravenously on day 1, and capecitabine (2000 mg/m^2^/day) orally on days 1–14, every 3 weeks.
  2. Combined use of bevacizumab with mFOLFOX6: 5 mg/kg bevacizumab, l-leucovorin (LV; 200 mg/m^2^), oxaliplatin (85 mg/m^2^), and bolus 5-FU (400 mg/m^2^), followed by infusion of 5-FU (2400 mg/m^2^) for 46 h every 2 weeks.
- Platinum Group.
  1. CAPOX, mFOLFOX6: same as above.
  2. SP: tegafur, gimeracil, and oteracil potassium (S-1) (80 mg/m^2^/day) orally on days 1–14 and cisplatin (60 mg/m^2^) intravenously on day 8 every 5 weeks.
  3. SOX: oxaliplatin (100 mg/m^2^) intravenously on day 1 and S-1 (80 mg/m^2^/day) orally on days 1–14 every 3 weeks.
  4. Monotherapy Group.
  5. S-1 (80 mg/m^2^/day) orally for 28 days every 6 weeks.
  6. Capecitabine (1250 mg/m^2^/day) orally for 14 days every 3 weeks.
  7. Uracil and tegafur (UFT; 300 mg/m^2^/day) orally for 28 days every 5 weeks.
  8. Gemcitabine (GEM; 1000 mg/m^2^) intravenously on days 1, 8, and 15 every 4 weeks.
  9. 5-FU+ LV: 5-FU (600 mg/m^2^) bolus plus LV (250 mg/m^2^) once a week for 6 weeks every 8 weeks.
  10. Docetaxel (DTX; 60 mg/m^2^) intravenously on day 1 every 3 weeks.

### Immunohistochemistry

Paraffin blocks or unstained slides were collected at the Department of Gastroenterology and Hepatology, Osaka University Graduate School of Medicine. All specimens were fixed in formalin, embedded in paraffin, and cut into 4-μm thick sections for immunohistochemistry (IHC) and haematoxylin and eosin staining. The primary antibodies for IHC are listed in Supplemental Table 1. Staining was conducted on the Dako Autostainer Link 48 platform (Agilent, Santa Clara, CA, USA) with an automated staining protocol. Immunohistochemically stained slides were independently evaluated by 2 of 3 certified gastroenterologists (T.A., T.T., and S.I.) who were blind to the clinicopathological information, and cases with different interpretations were assessed by a certified pathologist (E.M.). CD10 was expressed in a cytoplasmic pattern with membranous accentuation. MUC2, MUC5AC, MUC6, VEGF-A, and β-catenin were expressed in the cytoplasm of the tumour cells (Supplemental Figure 1a). TP53 and Ki67 were expressed in the nucleus of the tumour cells (Supplemental Figure 1a). Immunohistochemically stained slides were evaluated as follows: CD10, MUC2, MUC5AC, MUC6, and β-catenin were evaluated as positive if more than 5% of the tumour cells were stained, and VEGF-A, TP53, and Ki67 were evaluated as high if over 50% of the tumour cells were stained. Mismatch repair (MMR) protein (MLH1, MSH2, MSH6, and PMS2) was evaluated as negative when all tumour cells showed loss of nuclear staining compared with infiltrating lymphocytes as a positive internal control (Supplemental Figure 1b) and tumours with loss of any MMR protein were labelled as MMR protein-deficient (MMRD).

### Mucinous immunophenotypic classification

According to combined CD10 and mucinous immunophenotypes, we classified all cases, as shown in Supplemental Table 2, as intestinal type (I-type, CD10+ or MUC2+/MUC5AC−/MUC6-), gastrointestinal type (GI-type, CD10+ or MUC2+/MUC5AC+ or MUC6+), gastric type (G-type, CD10−/MUC2−/MUC5AC+ or MUC6+), or null type (N-type, CD10−/MUC2−/MUC5AC−/MUC6-), as previously reported for the duodenum [25], GC [26], and CRC [20].

### Statistical analysis

Continuous variables are presented as the median and interquartile range. Categorical valuables are presented as frequencies. Differences in the distribution of variables were evaluated using Fisher’s exact test. PFS and OS were estimated by the Kaplan-Meier method using the log-rank test. The hazard ratio (HR) and corresponding 95% confidence interval (CI) were estimated by univariate and multivariate Cox proportional hazards models with stratification variables and other relevant covariates (immunohistochemical expression and immunophenotypes). Variables determined to be significant in the univariate analysis were selected for the multivariate analysis. All reported *P*-values were 2-sided, and *P* < .05 was considered statistically significant. Statistical analyses were performed using JMP statistical software (version 14.3.0; SAS Institute, Inc., Cary, NC, USA).

## Results

### Clinicopathological characteristics

A total of 75 patients with mSBA were included in the study and 1 patient was excluded from the study due to insufficient SBA material for the analysis. Clinicopathological characteristics of the 74 remaining patients with mSBA are provided in Table 1. Of the 74 patients, 45 (60.8%) were older than 65 years of age, 49 (66.2%) were men, and 50 patients (67.6%) were PS 0. Primary tumour location was the duodenum in 38 patients (51.3%), jejunum in 27 (36.5%), and ileum in 9 (12.2%). The histological type of mSBA was differentiated-type in 57 patients (77.0%). The proportions of clinicopathological characteristics were comparable between patients with mDJA and those with mIA. The number of patients receiving each type of first-line chemotherapy is shown in Supplemental Table 3. Of the 74 cases, 16 (21.6%), 39 (52.7%), and 19 (25.7%) were classified into the Bevacizumab+ Platinum, Platinum, and Monotherapy Groups, respectively.

**Table 1:** Clinicopathological characteristics of patients with mSBA and comparison of the characteristics between the primary tumour location in the duodenum/ jejunum and ileum

Primary tumour location	Total	Duodenum/ Jejunum	Ileum	*P* value
All, n	74	65 (38/ 27)	9	
Sex (male), n (%)	49 (66.2)	44 (67.7)	5 (55.6)	0.475
Age > 65 years, n (%)	45 (60.8)	42 (64.6)	3 (33.3)	0.141
PS 0 or 1, n (%)	67 (90.5)	58 (89.2)	9 (100.0)	0.586
Complication of cancer in another organ, n (%)	21 (28.8)	19 (29.2)	2 (25.0)	1.000
Histological type (differentiated), n (%)	57 (77.0)	50 (76.9)	7 (77.8)	1.000
Number of metastatic organs > 2, n (%)	18 (24.3)	15 (23.1)	3 (33.3)	0.679
Metastasis site				
Liver, n (%)	26 (35.1)	23 (35.4)	3 (33.3)	1.000
Lung, n (%)	5 (6.8)	4 (6.2)	1 (11.1)	0.487
Lymph node, n (%)	21 (28.4)	18 (27.7)	3 (33.3)	0.707
Peritoneal dissemination, n (%)	25 (33.8)	21 (32.3)	4 (44.4)	0.475
Resection of primary tumour, n (%)	41 (55.4)	33 (50.8)	8 (88.9)	0.037
Post-operative recurrence, n (%) ^a^	9 (12.2)	7 (10.8)	2 (22.2)	0.299
CEA > 5 ng/ml, n (%) (*n* = 73)	35 (48.0)	29 (45.3)	6 (66.7)	0.296
CA19–9 > 37 U/ml, n (%) (*n* = 73)	34 (46.6)	31 (48.4)	3 (33.3)	0.489

*PS* performance status, *CEA* serum carcinoembryonic antigen, *CA19–9* carbohydrate antigen 19–9, ^a^ Patients who developed metastatic lesion after non-curative resection

### Immunohistochemical expression

Immunohistochemical expression data from the 74 patients with mSBA are shown in Table 2. Specimens were obtained by biopsy in 35 patients (47.3%) and by surgery in 39 patients (52.7%). Expression of VEGF-A was high in 42 patients (56.8%). Expression of CD10, MUC2, MUC5AC, and MUC6 was evaluated as positive in 55 (74.3%), 59 (79.7%), 45 (60.8%), and 29 (39.2%) of the patients, respectively. On the basis of mucinous immunophenotyping, 23 patients (31%) were classified as having I-type, 45 (60.8%) as having GI-type, 5 (6.8%) as having G-type, and 1 (1.4%) as having N-type of mSBA. The percentage of patients with I-type was significantly lower in those with mDJA (24.6%) than in those with mIA (77.8%, *P* = 0.003), and conversely, GI-type was significantly higher in those with mDJA (66.2%) than in those with mIA (22.2%, *P* = 0.023).

**Table 2:** Immunohistochemical molecular marker expression in patients with mSBA and comparison of the expression between the primary tumour location in duodenum/jejunum and the ileum

Primary tumour location	Total	Duodenum/ Jejunum	Ileum	*P* value
All, n	74	65 (38/ 27)	9	
Specimens (biopsy), n (%)	35 (47.3)	34 (52.3)	1 (11.1)	0.030
VEGF-A (high), n (%)	42 (56.8)	39 (60.0)	3 (33.3)	0.162
CD10 (positive), n (%)	55 (74.3)	48 (73.9)	7 (77.8)	1.000
MUC2 (positive), n (%)	59 (79.7)	50 (76.9)	9 (100.0)	0.189
MUC5AC (positive), n (%)	45 (60.8)	43 (66.2)	2 (22.0)	0.023
MUC6 (positive), n (%)	29 (39.2)	29 (44.6)	0 (0.0)	0.009
Mucinous immunophenotype				
Intestinal type, n (%)	23 (31.1)	16 (24.6)	7 (77.8)	0.003
Gastrointestinal type, n (%)	45 (60.8)	43 (66.2)	2 (22.2)	0.023
Gastric type, n (%)	5 (6.8)	5 (7.7)	0 (0.0)	1.000
Null type, n (%)	1 (1.4)	1 (1.5)	0 (0.0)	1.000
TP53 (high), n (%)	32 (43.2)	27 (41.5)	5 (55.6)	0.487
Ki67 (high), n (%)	56 (75.7)	50 (76.9)	6 (66.7)	0.679
β-catenin (positive), n (%)	9 (12.2)	8 (12.3)	1 (11.1)	1.000
MMRD, n (%)	4 (5.4)	3 (4.6)	1 (11.1)	0.411

*mSBA* metastatic small bowel adenocarcinoma, *VEGF-A* vascular endothelial growth factor A, *MMRD* mismatch repair protein deficiency

### Efficacy of bevacizumab-containing chemotherapy for patients with mSBA

The efficacy of bevacizumab-containing chemotherapy was investigated by stratifying patients into those with mDJA or mIA. In those with mIA, the OS in the Bevacizumab+ Platinum Group (51 months [19–94]) was significantly longer than that in the Platinum Group (17.5 months [12–23], *P* = 0.047; Supplemental Figure 2 b), as previously reported [15]. We also found that in those with mDJA, both the PFS and OS in the Bevacizumab+ Platinum Group (15 months [1-] and 26 months [5-]) tended to be longer than those in the Platinum Group (7 [5–9] and 17 [8–22], *P* = 0.075 and *P* = 0.077; Supplemental Figure 2c and d, respectively).

### VEGF-A expression as a factor for prolonging PFS and OS in patients with mDJA

When we searched for factors associated with a prolonged PFS and OS in mDJA, univariate analysis followed by multivariate analysis revealed that high VEGF-A expression was a significant factor for prolonging PFS (HR, 0.58, 95% CI, 0.34–0.99; Table 3) and a possible factor for prolonging OS (HR, 0.56, 95%CI, 0.31–1.01; Supplemental Table 4). The clinicopathological characteristics and immunohistochemical expression were not significantly different between patients with high VEGF-A expression and those with low VEGF-A expression (Supplemental Table 5).

**Table 3:** Univariate and multivariate analyses of immunohistochemical expression, mucinous immunophenotypes, and chemotherapy for prolonging PFS in patients with mDJA

		Univariate analysis			Multivariate analysis		
Variables	N	HR	95% CI	*P* value	HR	95% CI	*P* value
**VEGF-A (high)**	39	0.54	0.32–0.93	0.027	0.58	0.34–0.99	0.049
**CD10 (positive)**	48	0.77	0.43–1.39	0.396			
**MUC2 (positive)**	50	0.57	0.31–1.06	0.078			
**MUC5AC (positive)**	43	1.35	0.76–2.38	0.293			
**MUC6 (positive)**	29	1.50	0.88–2.57	0.134			
**I-type**	16	0.50	0.26–0.97	0.040	0.69	0.34–1.40	0.308
**GI-type**	43	1.33	0.75–2.35	0.316			
**G-type**	5	2.38	0.93–6.05	0.068			
**TP53 (high)**	27	0.75	0.44–1.29	0.307			
**Ki67 (high)**	50	0.50	0.27–0.92	0.026	0.66	0.34–1.25	0.208
**β-catenin (positive)**	8	0.87	0.37–2.05	0.759			
**MMRD**	3	1.38	0.33–3.00	0.652			
**Bevacizumab-containing** **chemotherapy**^**a**^	10	0.43	0.18–1.01	0.054	0.61	0.25–1.49	0.285
**Platinum-based chemotherapy**^**b**^	47	0.59	0.33–1.05	0.074	0.69	0.37–1.27	0.241

*PFS* progression-free survival, *mDJA* metastatic duodenal and jejunal adenocarcinoma, *CI* confidence interval, *HR* hazard ratio, *VEGF-A* vascular endothelial growth factor A, *I-type* intestinal type, *GI-type* gastrointestinal type, *G-type* gastric type, *MMRD* mismatch repair protein deficiency, ^a^ The reference is “Chemotherapy without bevacizumab”, ^b^ The reference is “Monotherapy”

**Fig. 1:**
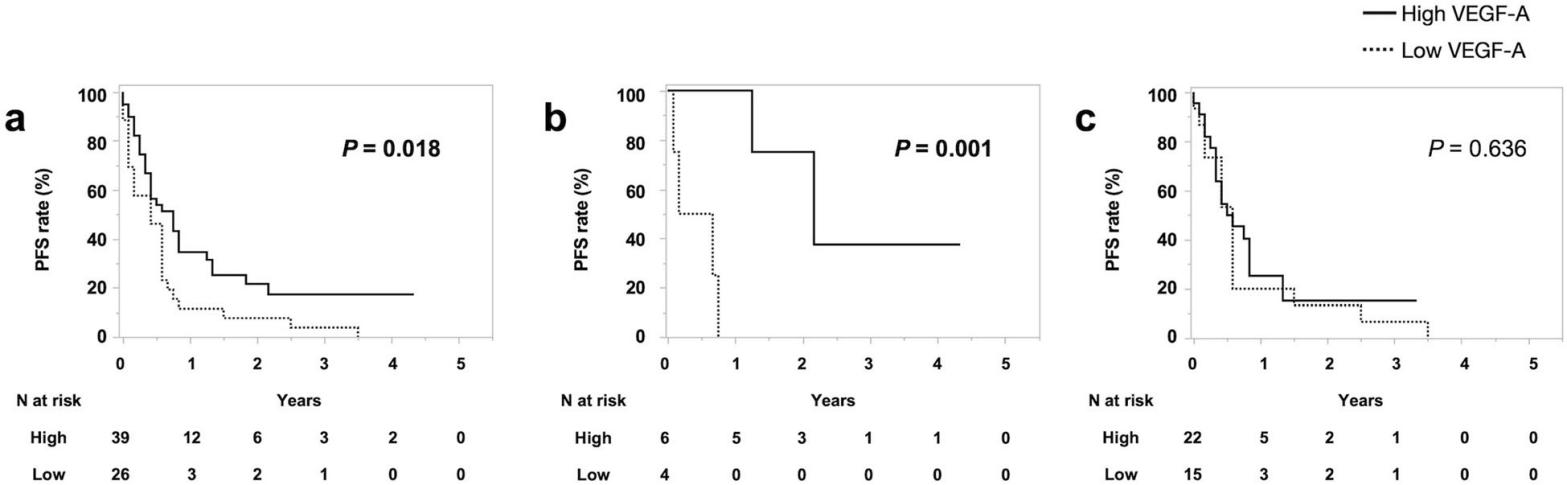
Cumulative PFS curve of mDJA patients with high VEGF-A expression or low VEGF-A expression (**a**) in the Bevacizumab+ Platinum Group (**b**) and in the Platinum Group (**c**). The PFS was significantly longer in mDJA patients with high VEGF-A expression (median [95%CI] 9 months [4–10]) than in those with low VEGF-A expression (5 months [1–7], *P* = 0.018) (**a**). In the Bevacizumab+ Platinum Group, the PFS was significantly longer in mDJA patients with high VEGF-A expression (26 months [15-]) than in those with low VEGF-A expression (5 months [1–9], *P* = 0.001) (**b**). In the Platinum Group, the PFS was significantly longer in mDJA patients with high VEGF-A expression (6.5 months [4–10]) than in those with low VEGF-A expression (7 months [2–7], *P* = 0.636) (**c**). PFS: progression-free survival, mDJA: metastatic duodenal and jejunal adenocarcinoma, VEGF-A: vascular endothelial growth factor A

We then investigated the PFS and the OS among mDJA patients with high or low VEGF-A expression. The PFS was significantly longer in patients with high VEGF-A expression (median [95% CI] 9 months [4–10]) than in those with low VEGF-A expression (5 months [1–7], *P* = 0.018; Fig. 1a) and the OS tended to be longer in those with high VEGF-A expression (20 months [15–24]) than in those with low VEGF-A expression (7 months [5–14], *P* = 0.059; Supplemental Figure 3a). In the Bevacizumab+ Platinum Group, the PFS was significantly longer in patients with high VEGF-A expression (26 months [15-]) than in those with low VEGF-A expression (5 months [1–9], *P* = 0.001; Fig. 1b) and the OS tended to be longer in patients with high VEGF-A expression than in those with low VEGF-A expression (*P* = 0.062; Supplemental Figure 3b). In the Platinum Group, neither the PFS nor the OS differed significantly between patients with high VEGF-A expression (6.5 months [4–10] and 18 months [11–22]) and patients with low VEGF-A expression (7 months [2–7] and 11 months [4–41], *P* = 0.636 and *P* = 0.482; Fig. 1c and Supplemental Figure 3c).

### VEGF-A expression and bevacizumab treatment for patients with mDJA

**Fig. 2:**
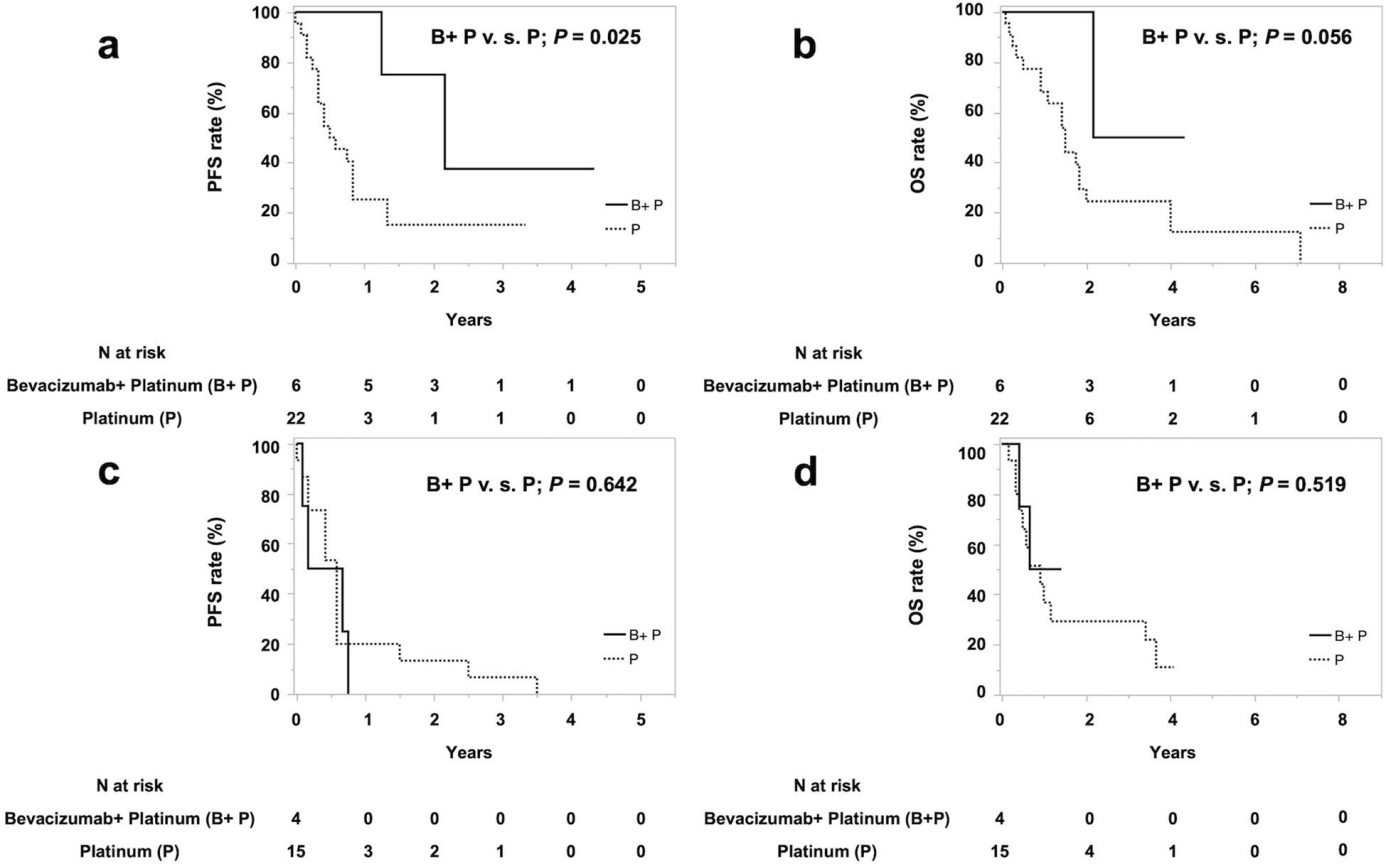
Cumulative PFS curve (**a**) and OS curve (**b**) of mDJA patients with high VEGF-A expression and cumulative PFS curve (**c**) and OS curve (**d**) of patients with low VEGF-A expression in the Bevacizumab+ Platinum (B+ P), Platinum (P) groups. In mDJA patients with high VEGF-A expression, the PFS was significantly longer in the B+ P group (median [95%CI] 26 months [15-]) than in the P group (6.5 months [4–10]; *P* = 0.025) (**a**). The OS tended to be longer in the B+ P group than in the P group (*P* = 0.056) (**b**). In mDJA patients with low VEGF-A expression, neither the PFS (**c**) nor the OS (**d**) was significantly longer in the B+ P group than in the P group (*P* = 0.519 and *P* = 0.642, respectively). PFS: progression-free survival, OS: overall survival, mDJA: metastatic duodenal and jejunal adenocarcinoma, VEGF-A: vascular endothelial growth factor A

We next investigated the PFS and OS among the treatment groups by stratifying patients with mDJA into groups with high or low VEGF-A expression. In patients with high VEGF-A expression, the PFS was significantly longer in the Bevacizumab+ Platinum Group (26 months [15-]) than in the Platinum Group (6.5 months [4–10], *P* = 0.025; Fig. 2a). In addition, the OS tended to be longer in the Bevacizumab+ Platinum Group than in the Platinum Group (*P* = 0.056; Fig. 2b). In patients with low VEGF-A expression, neither the PFS nor the OS differed significantly between the Bevacizumab+ Platinum and Platinum Groups (*P* = 0.519 and *P* = 0.642; Fig. 2c, d).

### Toxicity

Finally, patients were evaluated in terms of treatment-related toxicity. The proportion of patients with Grade 3 to 4 toxicity did not differ significantly between the Bevacizumab+ Platinum Group (50.0%) and Platinum Group (35.9%, *P* = 0.375; Table 4). The proportion tended to be smaller (16.7%) in the Monotherapy Group than in the Platinum Group (*P* = 0.214).

**Table 4:** Grade 3 to 4 toxicities during chemotherapy in the 3 treatment groups

Treatment Group	Bevacizumab+ Platinum	Platinum (reference)	Monotherapy
All toxicities, n (%)	8 (50.0)	14 (35.9)	3 (15.8)
Peripheral neuropathy, n (%)	3 (18.8)	5 (12.8)	0 (0.0)
Neutropenia, n (%)	2 (12.5)	8 (20.5)	2 (10.5)
General fatigue, n (%)	2 (12.5)	1 (2.6)	0 (0.0)
Gastrointestinal perforation, n (%)	0 (0.0)	1 (2.6)	0 (0.0)

## Discussion

To the best of our knowledge, this is the first study to evaluate the relation of the immunohistochemical expression of VEGF-A, which could be applied in clinical practice, to the efficacy of treatment with bevacizumab in combination with platinum-based first-line chemotherapy for patients with mDJA. A strength of the present study is that the immunostaining was centrally performed with an automated staining protocol and central reading in a multicentre setting. Although the multivariate analysis in Table 3 suggest that high expression of VEGF-A would be the prognostic factor but not the chemotherapy with bevacizumab, we also demonstrated in Fig. 1b and c that the PFS was significantly longer in patients with high VEGF-A expression than in those with low VEGF-A expression in the Bevacizumab+ Platinum group, but not in the Platinum group. These data suggest that the clinical value of bevacizumab in mDJA can be demonstrated when immunohistochemical VEGF-A is high. Furthermore, the results in Fig. 2 demonstrated that patients with mDJA having high VEGF-A expression who received platinum-based chemotherapy with bevacizumab as a first-line treatment had longer PFS and OS than those without bevacizumab. On the other hand, neither the PFS nor the OS of patients with low VEGF-A expression differed significantly between those treated with or without bevacizumab. The potential for immunohistochemical expression of VEGF-A to serve as a molecular biomarker for selecting bevacizumab-containing chemotherapy for patients with mDJA has not been evaluated previously. Thus, we first demonstrated that immunohistochemical expression of VEGF-A has potential as a biomarker for predicting the efficacy of bevacizumab-containing first-line chemotherapy in patients with mDJA.

The tumorigenesis of SBA reportedly differs from that of CRC in some aspects despite their morphological similarities. Immunohistochemical investigation of tumorigenic pathways in SBA and CRC revealed that positive β-catenin expression is less frequent in SBA (19.2 to 19.6%) than in CRC (78.6%), although the proportion of patients with high TP53 expression in SBA (41.6 to 53.8%) is similar to that in CRC (43.5%) and the proportion of patients with MMRD in SBA (8.0 to 23.0%) is similar to that in CRC (12.5%) [27–29]. The proportions of mSBA patients with positive β-catenin expression (12.1%), high TP53 expression (43.2%), and MMRD (5.4%) in our study were similar to those in previous reports [27, 29, 30]. We analysed each immunohistochemical expression separately in patients with mDJA or mIA. The proportion of mSBA patients with high TP53 expression, high Ki67 expression, positive β-catenin expression and MMRD did not differ between those with mDJA and mIA, consistent with a previous report [29]. Our data indicated that the expression of neither TP53, Ki67, β-catenin, nor MMRD was a factor for prolonging OS or PFS in patients with mSBA.

In the present study, we first evaluated the mucinous immunophenotype according to the expression of MUC2, MUC5AC, MUC6, and CD10 in patients with mSBA excluding the ampulla of Vater. Previous mucinous immunophenotypic classifications of GC and CRC revealed that the proportion of I-type is 10 to 30% in GC and 55 to 75% in CRC, and that of GI/G-type is 55 to 80% in GC and 5 to 30% in CRC [19, 20, 26, 31, 32]. In the present study, the proportions of I- (77.8%), GI- (22.2%), and G-type (0.0%) mIA were similar to those in CRC, while those of I- (24.6%), GI- (66.2%), and G-type (7.7%) mDJA were similar to those in GC. This finding indicates that application of a suitable chemotherapy regimen depending on the primary tumour location can be useful in mSBA.

Although VEGF-A is reported to have a key role in carcinogenesis, and its expression is related to the prognosis in SBA as well as CRC [16, 33], there are no reports regarding the usefulness of evaluating VEGF-A expression for selecting bevacizumab-containing chemotherapy in patients with mSBA. Our data revealed that VEGF-A expression was a predictive factor for the efficacy of bevacizumab for mDJA, as previously reported for upper gastrointestinal cancers, including metastatic GC [17]. For patients with mIA, only 9 patients were included in the present study and we could not evaluate whether VEGF-A expression was useful for selecting bevacizumab-containing chemotherapy in this group.

The present study has several limitations. First, this study was a retrospective study with a small sample size, and a patient selection bias cannot be excluded. Considering that mSBA is a very rare disease, however, this was one of the largest studies evaluating the clinical efficacy of bevacizumab in combination with platinum-based first-line chemotherapy in patients with mSBA. Although we demonstrated the potential of VEGF-A as an immunohistochemical biomarker for selecting bevacizumab-containing first-line chemotherapy with mDJA, the validation study is required to evaluate the result of this study due to the small number of patients. Second, the chemotherapy regimens were not unified because definite regimens have not been approved for mSBA and selection of the regimen was determined by each treating physician. Larger prospective studies are needed to determine the optimal cytotoxic chemotherapy regimen with bevacizumab as the first-line therapy in these patients.

## Conclusions

Immunohistochemical expression of VEGF-A has potential as a useful biomarker for predicting the efficacy of bevacizumab-containing first-line chemotherapy in patients with mDJA.

## Supplementary Information

**Additional file 1: Supplemental Table 1.** Antibodies used in the present study. **Supplemental Table 2.** Mucinous immunophenotypic classification in the present stud. **Supplemental Table 3.** First-line chemotherapy regimens used in 74 patients with mSBA. **Supplemental Table 4.** Univariate and multivariate analyses of immunohistochemical expression, mucinous immunophenotypes, and chemotherapy for prolonging OS in patients with mDJA. **Supplemental Table 5.** Comparison of clinicopathological characteristics and immunohistochemical expression of mDJA patients with high and low VEGF-A expression.

**Additional file 2: Supplemental Figure 1.** Molecular marker expression profile of CD10, mucins, VEGF-A, TP53, Ki67, β-catenin, and MMRD. (a) CD10 was expressed in a cytoplasmic pattern with membranous accentuation, and MUC2, MUC5AC, and MUC6 were expressed in the cytoplasm. VEGF-A, TP53, and Ki67 were expressed in the cytoplasm, and β-catenin was expressed in the nuclei. (b) When MLH1 was deficient, staining for MLH1 and PMS2 was negative and staining for MSH2 and MSH6 was positive. VEGF-A: vascular endothelial growth factor A. **Supplemental Figure 2.** Cumulative PFS curve (a) and OS curve (b) of mIA patients and cumulative PFS curve (c) and OS curve (d) of mDJA patients in the Bevacizumab+ Platinum (B+ P) Group, the Platinum (P) Group, and the Monotherapy (M) Group. In mIA patients, the PFS was longer in the B+ P Group (median [95%CI] 17.5 months [5–33]) than in the P Group (7 months [6–8]; *P* = 0.238) (a). The OS was significantly longer in the B+ P Group (51 months [19–94]) than in the P Group (17.5 months [12–23]; *P* = 0.047) (b). In mDJA patients, the PFS did not differ significantly between the B+ P Group (15 months [1-]) and the P Group (7 months [5–9]; *P* = 0.075) (c). The OS was significantly longer in the B+ P Group (26 months [5-]) than in the P Group (17 months [8–22]; *P* = 0.077) (d). PFS: progression-free survival, OS: overall survival, mIA: metastatic ileal adenocarcinoma, mDJA: metastatic duodenal and jejunal adenocarcinoma. **Supplemental Figure 3.** Cumulative OS curve of mDJA patients with high VEGF-A expression or low VEGF-A expression (a) in Bevacizumab+ Platinum (B+ P) Group (b) and in Platinum (P) Group (c). The OS tended to be longer in mDJA patients with high VEGF-A expression (median [95%CI] 20 months [15–24]) than in those with low VEGF-A expression (7 months [5–14], *P* = 0.059) (a). In B+ P Group, the OS tended to be longer in mDJA patients with high VEGF-A expression than in those with low VEGF-A expression (*P* = 0.062) (b). In P Group, the OS was significantly longer in mDJA patients with high VEGF-A expression (18 months [11–22]) than in those with low VEGF-A expression (11 months [4–41], *P* = 0.482) (c). OS: overall survival, mDJA: metastatic duodenal and jejunal adenocarcinoma, VEGF-A: vascular endothelial growth factor A.

## Data Availability

The datasets used and analysed during the current study are available from the corresponding author on reasonable request.

## Abbreviations

SBA: Small bowel adenocarcinoma
mSBA: Metastatic SBA
mIA: Metastatic ileal adenocarcinoma
mDJA: Metastatic duodenal and jejunal adenocarcinoma
VEGF: Vascular endothelial growth factor
GC: Gastric cancer
CRC: Colorectal cancer
I-type: Intestinal type
GI-type: Gastrointestinal type
G-type: Gastric type
N-type: Null type
PS: Performance status
CEA: Serum carcinoembryonic antigen
CA19–9: Carbohydrate antigen 19–9
PFS: Progression-free survival
OS: Overall survival
mFOLFOX6: Modified FOLFOX6
IHC: Immunohistochemistry
MMR: Mismatch repair
MMRD: Mismatch repair deficient
HR: Hazard ratio
CI: Confidence interval
