# Supplementary material for: Vascular endothelial growth factor-A is an Immunohistochemical biomarker for the efficacy of bevacizumab-containing chemotherapy for duodenal and jejunal adenocarcinoma
Source: BMC Cancer. 2021 Aug 31;21:978. doi: 10.1186/s12885-021-08724-5 (PMC8406832; doi:10.1186/s12885-021-08724-5)
Supplement: Supplementary file 1 — Additional file 1: Supplemental Table 1. Antibodies used in the present study. Supplemental Table 2. Mucinous immunophenotypic classification in the present stud. Supplemental Table 3. First-line chemotherapy regimens used in 74 patients with mSBA. Supplemental Table 4. Univariate and multivariate analyses of immunohistochemical expression, mucinous immunophenotypes, and chemotherapy for prolonging OS in patients with mDJA. Supplemental Table 5. Comparison of clinicopathological characteristics and immunohistochemical expression of mDJA patients with high and low VEGF-A expression. [file 12885_2021_8724_MOESM1_ESM.docx]

**Supplemental Table 1.** Antibodies used in the present study

| Antibody | Clone | Dilution | Supplier |
| --- | --- | --- | --- |
| CD10 | 56C6 | 1:100 | Leica Biosystems, Nussloch, Germany |
| MUC2 | Ccp58 | 1:400 | Santa Cruz Biotechnology, Dallas, United States |
| MUC5AC | CLH2 | 1:400 | Santa Cruz Biotechnology, Dallas, United States |
| MUC6 | CLH5 | 1:400 | Santa Cruz Biotechnology, Dallas, United States |
| VEGF-A | VG-1 | 1:400 | Agilent, Santa Clara, United States |
| TP53 | DO-7 | 1:500 | Leica Biosystems, Nussloch, Germany |
| Ki67 | MIB-1 | 1:100 | Agilent, Santa Clara, United States |
| β-catenin | β-Catenin-1 | 1:1600 | Agilent, Santa Clara, United States |
| MLH1 | ES05 | 1:100 | Leica Biosystems, Nussloch, Germany |
| MSH2 | FE11 | 1:100 | Agilent, Santa Clara, United States |
| MSH6 | EP49 | 1:800 | Agilent, Santa Clara, United States |
| PMS2 | EP51 | 1:100 | Agilent, Santa Clara, United States |

**Supplemental Table 2.** Mucinous immunophenotypic classification in the present study

| CD10, MUC2 | MUC5AC, MUC6 | Mucinous immunophenotype |
| --- | --- | --- |
| CD10+/MUC2+  or  CD10+/MUC2-  or  CD10-/MUC2+ | MUC5AC+/MUC6+  or  MUC5AC+/MUC6-  or  MUC5AC-/MUC6+ | Gastrointestinal type |
|  |  |  |
|  | MUC5AC-/MUC6- | Intestinal type |
|  |  |  |
| CD10-/MUC2- | MUC5AC+/MUC6+  or  MUC5AC+/MUC6-  or  MUC5AC-/MUC6+ | Gastric type |
|  | MUC5AC-/MUC6- | Null type |

**Supplemental Table 3.** First-line chemotherapy regimens used in 74 patients with mSBA

| Treatment Group | All | Bevacizumab+ Platinum | Platinum | Monotherapy |
| --- | --- | --- | --- | --- |
| All, n | 74 | 16 | 39 | 19 |
| CAPOX, n (%) | 22 (29.7) | 8 (50.0) | 14 (35.9) |  |
| mFOLFOX6, n (%) | 21 (28.4) | 8 (50.0) | 13 (33.3) |  |
| S-1, Capecitabine or UFT, n (%) | 13 (17.6) |  |  | 13 (68.4) |
| SP, n (%) | 9 (12.2) |  | 9 (23.1) |  |
| GEM, n (%) | 4 (5.4) |  |  | 4 (21.1) |
| SOX, n (%) | 3 (4.1) |  | 3 (7.7) |  |
| 5-FU+ LV, n (%) | 1 (1.3) |  |  | 1 (5.3) |
| DTX, n (%) | 1 (1.3) |  |  | 1 (5.3) |

mSBA: metastatic small bowel adenocarcinoma, CAPOX: capecitabine and oxaliplatin, mFOLFOX6: 5-fluorouracil, L-leucovorin (LV), and oxaliplatin, S-1: tegafur, gimeracil, and oteracil potassium, UFT: uracil and tegafur, SP: S-1 and cisplatin, SOX: S-1 and oxaliplatin, 5-FU+ LV: 5-fluorouracil and LV, DTX: docetaxel.

**Supplemental Table 4.** Univariate and multivariate analyses of immunohistochemical expression, mucinous immunophenotypes, and chemotherapy for prolonging OS in patients with mDJA.

|  |  | **Univariate analysis** | | | **Multivariate analysis** | | |
| --- | --- | --- | --- | --- | --- | --- | --- |
| **Variables** | N | HR | 95% CI | *P* value | HR | 95% CI | *P* value |
| **VEGF-A (high)** | 39 | 0.58 | 0.32-1.04 | 0.067 | 0.56 | 0.31-1.01 | 0.056 |
| **CD10 (positive)** | 48 | 0.66 | 0.36-1.23 | 0.194 |  |  |  |
| **MUC2 (positive)** | 50 | 0.74 | 0.38-1.47 | 0.404 |  |  |  |
| **MUC5AC (positive)** | 43 | 1.07 | 0.59-1.96 | 0.808 |  |  |  |
| **MUC6 (positive)** | 29 | 1.20 | 0.68-2.13 | 0.518 |  |  |  |
| **I-type** | 16 | 0.59 | 0.29-1.19 | 0.141 |  |  |  |
| **GI-type** | 43 | 1.11 | 0.60-2.02 | 0.728 |  |  |  |
| **G-type** | 5 | 2.28 | 0.89-5.81 | 0.084 | 1.65 | 0.63-4.34 | 0.303 |
| **TP53 (high)** | 27 | 0.65 | 0.36-1.17 | 0.153 |  |  |  |
| **Ki67 (high)** | 50 | 0.59 | 0.31-1.14 | 0.119 |  |  |  |
| **β-catenin (positive)** | 8 | 0.86 | 0.36-2.33 | 0.867 |  |  |  |
| **MMRD** | 3 | 0.79 | 0.19-3.33 | 0.757 |  |  |  |
| **Bevacizumab-containing**  **Chemotherapy *** | 10 | 0.31 | 0.09-1.02 | 0.054 | 0.39 | 0.12-1.31 | 0.130 |
| **Platinum-based chemotherapy **** | 47 | 0.54 | 0.29-0.98 | 0.044 | 0.61 | 0.32-1.15 | 0.131 |

OS: overall survival, mDJA: metastatic duodenal and jejunal adenocarcinoma, CI: confidence interval, HR: hazard ratio, I-type: intestinal type, GI-type: gastrointestinal type, G-type: gastric type, MMRD: mismatch repair protein deficient, VEGF-A: vascular endothelial growth factor A, *: The reference is “Chemotherapy without bevacizumab”, **: The reference is “Monotherapy”

**Supplemental Table 5.** Comparison of clinicopathological characteristics and immunohistochemical expression of mDJA patients with high and low VEGF-A expression.

| VEGF-A expression | High | Low | *P* value |
| --- | --- | --- | --- |
| All, n | 39 | 26 |  |
| Primary tumour location (Duodenum), n (%) | 22 (56.4) | 16 (61.5) | 0.798 |
| Male, n (%) | 27 (69.2) | 17 (65.4) | 0.791 |
| Age >65 years, n (%) | 24 (61.5) | 18 (69.2) | 0.602 |
| PS 0 or 1, n (%) | 35 (89.7) | 23 (88.5) | 1.000 |
| Complication of cancer in another organ, n (%) | 11 (28.2) | 8 (30.7) | 1.000 |
| Histological type (differentiated), n (%) | 32 (82.1) | 18 (69.2) | 0.247 |
| Number of Metastatic organs > 2, n (%) | 7 (18.0) | 8 (30.8) | 0.247 |
| Metastasis site |  |  |  |
| Liver, n (%) | 13 (33.3) | 10 (38.5) | 0.671 |
| Lung, n (%) | 2 (5.1) | 2 (7.7) | 1.000 |
| Lymph node, n (%) | 10 (25.6) | 8 (30.8) | 0.650 |
| Peritoneal dissemination, n (%) | 12 (30.8) | 9 (34.6) | 0.745 |
| CEA >5ng/ml, n (%) (N=64) | 16 (42.1) | 13 (50.0) | 0.613 |
| CA19-9 >37U/ml, n (%) | 20 (52.6) | 11 (42.3) | 0.455 |
| Resection of primary tumour, n (%) | 21 (53.9) | 12 (46.2) | 0.617 |
| Post-operative recurrence, n (%) * | 5 (12.8) | 2 (7.7) | 0.692 |
| I-type, n (%) | 12 (30.7) | 4 (15.4) | 0.240 |
| GI-type, n (%) | 26 (66.7) | 17 (65.4) | 1.000 |
| G-type, n (%) | 1 (2.6) | 4 (15.4) | 0.148 |
| N-type, n (%) | 0 (0.0) | 1 (3.9) | 0.400 |
| TP53 (high), n (%) | 17 (45.6) | 10 (38.5) | 0.798 |
| Ki67 (high), n (%) | 31 (79.5) | 19 (73.1) | 0.563 |
| β-Catenin (positive), n (%) | 5 (12.8) | 1 (3.9) | 1.000 |
| MMRD, n (%) | 2 (5.1) | 1 (3.9) | 1.000 |
| Bevacizumab-containing chemotherapy, n (%) | 6 (15.4) | 4 (15.4) | 1.000 |
| Platinum-based chemotherapy, n (%) | 28 (71.8) | 19 (73.1) | 1.000 |

mDJA: metastatic duodenal and jejunal adenocarcinoma, VEGF-A: vascular endothelial growth factor A, PS: performance Status, CEA: carcinoembryonic antigen, CA19-9: carbohydrate antigen 19-9, I-type: intestinal type, GI-type: gastrointestinal type, G-type: gastric type, MMRD: mismatch repair protein deficiency, *: Patients who developed metastatic lesion after non-curative resection
