# Supplementary material for: Vascular endothelial growth factor-A is an Immunohistochemical biomarker for the efficacy of bevacizumab-containing chemotherapy for duodenal and jejunal adenocarcinoma
Source: BMC Cancer. 2021 Aug 31;21:978. doi: 10.1186/s12885-021-08724-5 (PMC8406832; doi:10.1186/s12885-021-08724-5)
Supplement: Supplementary file 2 — Additional file 2: Supplemental Figure 1. Molecular marker expression profile of CD10, mucins, VEGF-A, TP53, Ki67, β-catenin, and MMRD. (a) CD10 was expressed in a cytoplasmic pattern with membranous accentuation, and MUC2, MUC5AC, and MUC6 were expressed in the cytoplasm. VEGF-A, TP53, and Ki67 were expressed in the cytoplasm, and β-catenin was expressed in the nuclei. (b) When MLH1 was deficient, staining for MLH1 and PMS2 was negative and staining for MSH2 and MSH6 was positive. VEGF-A: vascular endothelial growth factor A. Supplemental Figure 2. Cumulative PFS curve (a) and OS curve (b) of mIA patients and cumulative PFS curve (c) and OS curve (d) of mDJA patients in the Bevacizumab+ Platinum (B+ P) Group, the Platinum (P) Group, and the Monotherapy (M) Group. In mIA patients, the PFS was longer in the B+ P Group (median [95%CI] 17.5 months [5–33]) than in the P Group (7 months [6–8]; P = 0.238) (a). The OS was significantly longer in the B+ P Group (51 months [19–94]) than in the P Group (17.5 months [12–23]; P = 0.047) (b). In mDJA patients, the PFS did not differ significantly between the B+ P Group (15 months [1-]) and the P Group (7 months [5–9]; P = 0.075) (c). The OS was significantly longer in the B+ P Group (26 months [5-]) than in the P Group (17 months [8–22]; P = 0.077) (d). PFS: progression-free survival, OS: overall survival, mIA: metastatic ileal adenocarcinoma, mDJA: metastatic duodenal and jejunal adenocarcinoma. Supplemental Figure 3. Cumulative OS curve of mDJA patients with high VEGF-A expression or low VEGF-A expression (a) in Bevacizumab+ Platinum (B+ P) Group (b) and in Platinum (P) Group (c). The OS tended to be longer in mDJA patients with high VEGF-A expression (median [95%CI] 20 months [15–24]) than in those with low VEGF-A expression (7 months [5–14], P = 0.059) (a). In B+ P Group, the OS tended to be longer in mDJA patients with high VEGF-A expression than in those with low VEGF-A expression (P = 0.062) (b). In P Group, the OS was si [file 12885_2021_8724_MOESM2_ESM.docx]

**
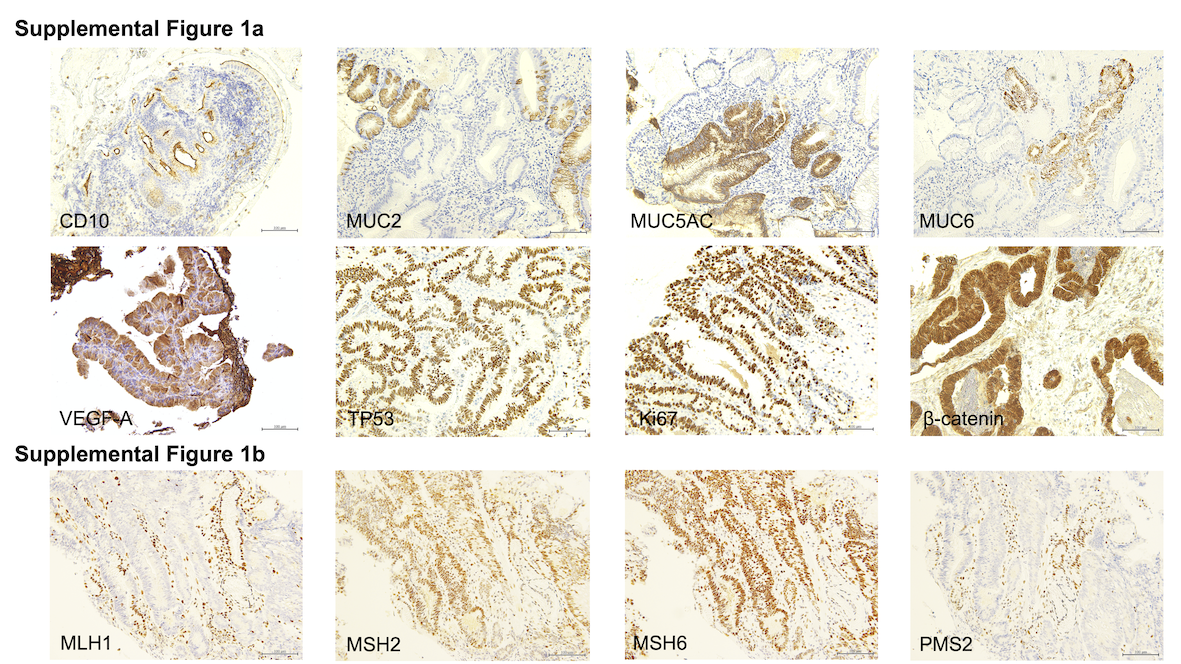
**

**Supplemental Figure 1.** Molecular marker expression profile of CD10, mucins, VEGF-A, TP53, Ki67, β-catenin, and MMRD. (a) CD10 was expressed in a cytoplasmic pattern with membranous accentuation, and MUC2, MUC5AC, and MUC6 were expressed in the cytoplasm. VEGF-A, TP53, and Ki67 were expressed in the cytoplasm, and β-catenin was expressed in the nuclei. (b) When MLH1 was deficient, staining for MLH1 and PMS2 was negative and staining for MSH2 and MSH6 was positive. VEGF-A: vascular endothelial growth factor A


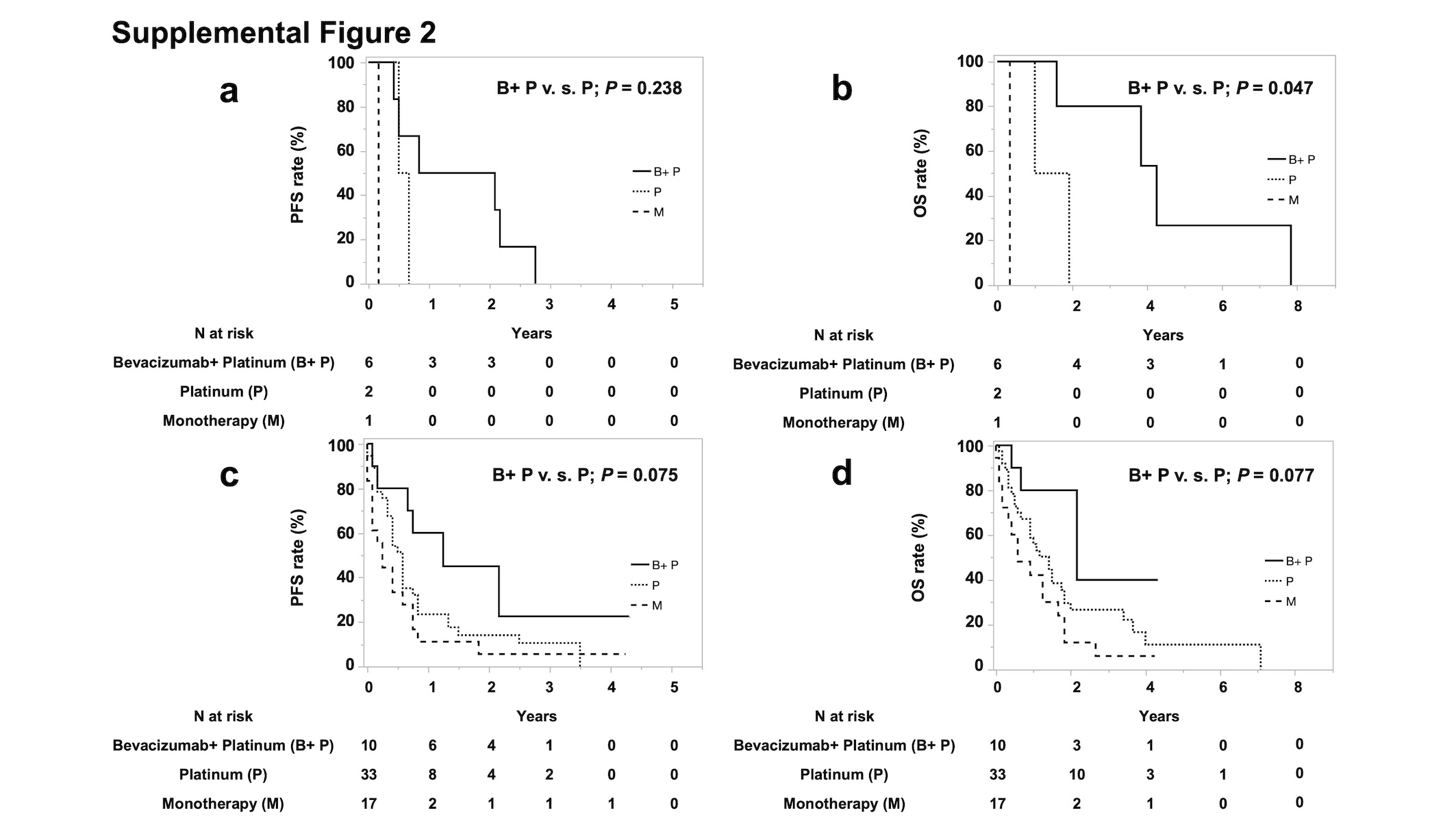


**Supplemental Figure 2.** Cumulative PFS curve (a) and OS curve (b) of mIA patients and cumulative PFS curve (c) and OS curve (d) of mDJA patients in the Bevacizumab+ Platinum (B+ P) Group, the Platinum (P) Group, and the Monotherapy (M) Group. In mIA patients, the PFS was longer in the B+ P Group (median [95%CI] 17.5 months [5-33]) than in the P Group (7 months [6-8]; *P* = 0.238) (a). The OS was significantly longer in the B+ P Group (51 months [19-94]) than in the P Group (17.5 months [12-23]; *P* = 0.047) (b). In mDJA patients, the PFS did not differ significantly between the B+ P Group (15 months [1-]) and the P Group (7 months [5-9]; *P* = 0.075) (c). The OS was significantly longer in the B+ P Group (26 months [5-]) than in the P Group (17 months [8-22]; *P* = 0.077) (d). PFS: progression-free survival, OS: overall survival, mIA: metastatic ileal adenocarcinoma, mDJA: metastatic duodenal and jejunal adenocarcinoma

**Supplemental Figure 3.** Cumulative OS curve of mDJA patients with high VEGF-A expression or low VEGF-A expression (a) in Bevacizumab+ Platinum (B+ P) Group (b) and in Platinum (P) Group (c). The OS tended to be longer in mDJA patients with high VEGF-A expression (median [95%CI] 20 months [15-24]) than in those with low VEGF-A expression (7 months [5-14], *P* = 0.059) (a). In B+ P Group, the OS tended to be longer in mDJA patients with high VEGF-A expression than in those with low VEGF-A expression (*P* = 0.062) (b). In P Group, the OS was significantly longer in mDJA patients with high VEGF-A expression (18 months [11-22]) than in those with low VEGF-A expression (11 months [4-41], *P* = 0.482) (c). OS: overall survival, mDJA: metastatic duodenal and jejunal adenocarcinoma, VEGF-A: vascular endothelial growth factor A
